# Green Coffee Defects and Their Aroma-Relevant Precursors: A Review of Classification Systems, Causal Pathways, and Sensory Chemistry

**DOI:** 10.3390/foods15213809

**Published:** 2026-10-24

**Authors:** Steffen Schwarz, Dirk W. Lachenmeier

**Affiliations:** 1 Coffee Consulate, Hans-Thoma-Straße 20, 68163 Mannheim, Germany; schwarz@coffee-consulate.com; 2 Chemisches und Veterinäruntersuchungsamt (CVUA) Karlsruhe, Weissenburger Str. 3, 76187 Karlsruhe, Germany

**Keywords:** green coffee, coffee defects, *Coffea*, aroma precursors, coffee roasting, quality classification, off-flavors, sensory chemistry

## Abstract

Green coffee defects are evaluated worldwide as visible abnormalities, physical damage, foreign matter, or off-odor, yet the relationship between grading label and cup quality is neither direct nor uniform. This review integrates international and national classification systems with evidence on defect formation, green bean composition, roasting chemistry, and sensory consequences. It shows that standards differ in sample mass, counting basis, legal status, and intended purpose, so their values cannot be converted into a common sensory scale. Defects are best understood as disturbances to precursor pools or as processing and safety risks; they are not fixed flavor entities. Compositional and volatile differences relative to sound coffee are documented for black, sour/brown, immature, insect-damaged, and aged beans; many other visible categories lack defect-specific odor-active or dose–response data. The term quaker illustrates the central nomenclature problem: standards commonly link it to immature beans, whereas a differentiated physiological position treats emaciated beans as a separate pathway. Rio flavor and potato taste defect provide more specific chemical signatures than most visual defects. A precursor-centered framework is proposed to connect grading with analytical and roasting practice while retaining uncertainty where the evidence chain is incomplete.

## 1. Introduction

Green coffee is traded, graded, and roasted as a biological raw material whose composition is set by fruit development and then altered by harvesting, processing, drying, storage, and mechanical handling. A defect is consequently not a single scientific entity: it may be a visually abnormal bean, a fragment of fruit tissue, a foreign object, an infestation sign, an odor detected in the green lot, or a cup fault revealed only after roasting and brewing. The International Organization for Standardization (ISO) standard ISO 10470 separates foreign matter, fruit components, irregularly formed beans, visually abnormal beans, and off-taste coffee, whereas ISO 4149 sets out an olfactory and visual examination for a laboratory sample before defects are separated and weighed [1,2]. This breadth is operationally useful, but it also means that the word “defect” does not by itself identify a causal mechanism, an analytical marker, or a sensory threshold.

A stone is a safety and equipment risk but has no coffee-derived aroma-precursor pathway; parchment or husk represents an incomplete separation outcome; a black bean may integrate fruit decay, microbial activity, and altered reserve composition; and a stinker may be visually inconspicuous despite a strong cup consequence. The Specialty Coffee Association of America (SCAA) handbook similarly distinguishes primary and secondary defects for a washed *Coffea arabica* grading system, but this operational hierarchy is not a chemical taxonomy and should not be interpreted as a ranking of universal cup intensity [3].

A useful scientific model is to separate four linked, but non-equivalent, levels. First, morphology describes the object visible in green coffee: color, shape, perforation, fragment size, or associated foreign material. Second, etiology describes the developmental, microbial, processing, or storage pathway that could have produced that appearance. Third, chemistry concerns non-volatile precursors, pre-existing volatile markers, and their changes upon roasting. Finally, sensory evidence concerns odor-active compounds, controlled mixtures, cup testing, or consumer perception. Volatile profiling can distinguish defective from sound beans, but a detected signal is not automatically an odor-active compound in the beverage and does not establish a sensory cause [4,5,6]. This review accordingly treats marker evidence and sensory proof as different evidential levels.

The need for that separation is clearest in roasting. Sucrose, free amino acids, chlorogenic acids (CGAs), trigonelline, lipids, and organic acids influence reaction routes and flavor formation, but their concentration differences do not map one-to-one onto a named cup defect. Maillard and Strecker chemistry transform nitrogenous and carbohydrate precursors during roasting, while lipid oxidation and microbial metabolites may introduce other pathways or suppress desirable aroma formation [7,8,9,10,11]. A defect can thus lower quality through a deficit of favorable precursors, an excess of compounds associated with deterioration, uneven heat transfer, or an extrinsic contaminant.

The literature is also complicated by language. Many terms have regional origins and were incorporated into grading systems with different scopes. “Ardido,” “sour,” “brown,” “*vinagre*,” and “fermented” overlap but are not necessarily coextensive; “shell,” “parchment,” and “husk” designate different plant materials; and “insect damaged” depends on each system’s threshold for holes or galleries. The term quaker is especially controversial. ISO 10470 presents “immature bean; ‘quaker’ bean” as one category, and the SCAA handbook likewise uses immature/unripe as a grading category [1,3]. By contrast, Coffee Consulate’s differentiated material uses quaker for a lightweight, shriveled, nutritionally or physiologically exhausted bean and keeps it separate from the immature, early-harvested bean [12]. These are competing definitions, not alternative labels that can safely be merged; the two usages are compared in detail in Section 5.5.

This review has three aims. First, it compares the classification architecture of national, international, and exchange systems without converting their original sample masses or defect values. Second, it organizes green coffee defects by likely stage of origin to clarify what a grading observation can and cannot imply about cause. Third, it reviews the evidence on composition, volatile markers, roasting transformations, and sensory relevance for the principal defect types, with special attention to the gaps between chemical discrimination and confirmed cup aroma. The focus is green coffee of *Coffea arabica* and *Coffea canephora*. The intended outcome is a more cautious precursor-centered interpretation of defects for researchers, graders, and roasters.

## 2. Materials and Methods

This article is a structured narrative review. Its normative basis comprises national, international, and exchange classification systems and standards: ISO 10470 and ISO 4149; the SCAA/Specialty Coffee Association (SCA) handbook; the Intercontinental Exchange (ICE) rules, namely the Coffee “C” Rules of ICE Futures U.S. and the Robusta Coffee Futures Contract of ICE Futures Europe; and national or regional systems from Brazil, Colombia, Peru, Mexico, Costa Rica, El Salvador, Nicaragua, East Africa, Uganda, Tanzania, Indonesia, Vietnam, and Papua New Guinea [1,2,3,13,14,15,16,17,18,19,20,21,22,23,24,25,26,27]. Selection of the national and regional systems reflected three considerations, not a production-volume ranking: coverage of the principal counting architectures in use (defect equivalents, *faltas*, point values, mass percentages, per-kilogram equivalents, and a volumetric-tray basis), coverage of major producing regions for both *Coffea arabica* and *Coffea canephora*, and the availability of the official text as a citable primary document. Origins not individually included remain accessible through the International Coffee Organization (ICO) compilations discussed in Section 3.2. These are read together with the vocabulary standard ISO 3509, the moisture-determination method ISO 6673, the SCA Coffee Value Assessment documents, and the ICO quality-improvement and harmonization documents [28,29,30,31,32]. The comparison was deliberately source-preserving: sample masses, defect-equivalent values, weight percentages, and system names are reported as specified by their documents, not normalized by conversion.

Scientific literature was identified in bibliographic databases and publisher platforms (PubMed/PMC, ScienceDirect, Wiley Online Library, SpringerLink, ACS Publications, Taylor & Francis Online, MDPI, and SciELO), in the ISO Online Browsing Platform and national standards portals, and on the official document servers of the ICO, the SCA, and ICE, covering publications up to August 2026 with no language restriction (sources in English, Portuguese, Spanish, German, Indonesian, and Vietnamese were consulted). The subject scope covered defective green coffee, green coffee storage, roasting chemistry, coffee fermentation, mycotoxins, coffee berry borer damage, Rio flavor, and potato taste defect. Searches used combinations of “green coffee defect,” “black bean,” “sour bean,” “immature coffee,” “quaker,” “coffee berry borer,” “coffee storage,” “ochratoxin A,” “Rio flavor,” “2,4,6-trichloroanisole,” “potato taste defect,” “2-isopropyl-3-methoxypyrazine,” “coffee volatile marker,” “GC-O,” and “coffee roasting precursor.” The keywords were used singly and in combination across these platforms without a start-date restriction. Because this is a narrative rather than a systematic review, per-database search protocols and hit counts were not recorded. Database retrieval was complemented by backward and forward citation chasing from the included publications, and the normative texts were obtained directly from the issuing organizations and not through bibliographic databases. This terminology reflects the vocabulary used by the normative documents and the core chemical literature and does not assume that regional terms are interchangeable [1,3,4,5,7]. The search and source-selection record is summarized in Table A1 (Appendix A).

Sources were assessed in a hierarchy appropriate to the question. Primary normative texts were preferred for definitions, sample bases, classification logic, and limits. Primary analytical or experimental studies were preferred for chemical compositions, volatile profiles, insect-associated changes, storage transformations, microbial associations, and sensory results. Reviews and technical institutional sources were used to frame mechanisms or identify gaps where primary evidence was not available. Non-verifiable trade claims, unsupported sensory descriptors, and sources that did not identify their material or method sufficiently for the claim being made were excluded. For example, volatile-marker reports were retained as evidence of analytical differentiation, whereas unverified claims that a single marker necessarily produces a named cup note were not carried forward [4,5,6].

The narrative synthesis was organized around a causal chain: origin stage, visible or olfactory classification, green bean composition, roasting-reaction potential, and sensory outcome. “Aroma precursor” is used here for a green bean constituent that may participate in roasting reactions or determine their balance; it is not used to imply that every difference in a precursor has a known flavor outcome. Similarly, a “marker volatile” denotes a compound useful for distinguishing sample groups unless odor activity, concentration relative to threshold, or controlled sensory evidence has been established. This distinction matters because defect-specific gas chromatography–olfactometry (GC-O), aroma extract dilution analysis, or recombination studies remain sparse for individual defects [4,7,10].

The review does not estimate defect prevalence, calculate pooled effect sizes, or infer a common quality score across standards. It also does not treat all coffee species, origins, processing methods, or roast degrees as interchangeable experimental backgrounds. Where a study is restricted to *Coffea arabica*, a particular processing regime, or a defined defect mixture, that restriction limits the generality of the interpretation. The term *Coffea canephora* is used throughout this review except in the official title “ICE Futures Robusta Coffee Futures Contract” and quoted grade terminology [14].

## 3. Defect Classification Systems Worldwide

### 3.1. International, Specialty, and Exchange Frameworks

ISO 10470 is a defect reference chart, not a trade-grade schedule. It assigns categories and coefficients for loss of mass and sensorial concern, so that defects are expressed through a quality-impact logic instead of the piece-equivalent system used in several commercial rules [1]. ISO 4149 complements that reference chart by specifying olfactory and visual examination and the determination of foreign matter and defects on a 300 g laboratory sample [2]. Taken together, these documents define a common descriptive language and examination procedure, but they do not establish a universal acceptance limit or declare that a coefficient is a measured sensory intensity. The examination standard was revised while this manuscript was in preparation: ISO 4149:2025 (Edition 3, published November 2025) supersedes the 2005 edition, and the companion moisture method was likewise revised as ISO 6673:2025 [2,29]. ISO 10470:2004 remains the current defect reference chart and is under systematic review [1]. The vocabulary for coffee and coffee products is set by ISO 3509, which cross-references ISO 10470 for defect categories; the defect terms themselves are defined in clause 3 of ISO 10470 [1,28].

The SCAA/SCA handbook (originally issued 2 April 2004, revised 26 April 2013; the edition cited here is the 2013 revision) serves a different purpose. It applies to washed *Coffea arabica* and uses a 350 g green sample with full defect equivalents, together with a 100 g roasted-coffee assessment for quakers [3]. Its primary/secondary distinction is influential in specialty practice: full black, full sour, dried cherry/pod, fungus-damaged material, and foreign matter are treated as primary defects, while partial black, partial sour, parchment, floater, immature/unripe, withered, shell, broken/chipped/cut, husk, and slight insect damage are secondary defects [3].

The SCA framework itself has moved on. The components of the SCA Coffee Value Assessment (CVA), SCA Standards 102 (sample preparation and tasting mechanics), 103 (descriptive assessment), and 104 (affective assessment), were adopted in November 2024 and, in the SCA’s own words, supersede the 2004 Cupping Protocol and Form [30,33,34,35]. Two consequences matter for this review. First, the CVA reduces the sensory defect list to three cup defects (moldy, phenolic, and potato), which is a far coarser sensory taxonomy than the visual one [35]. Second, the CVA Physical Assessment remains based on the 2004 green-grading system and is still in research and development, so the 2004/2013 defect logic reviewed here is superseded as a cupping protocol but not as a green-grading architecture [30].

Exchange rules add a further logic. They are contract-delivery specifications set by a private exchange operator, not standards, and they apply to coffee of many origins tendered against a futures contract. ICE Futures U.S. Coffee “C” uses a volumetric tray and counts full imperfections relative to an origin-specific basis rather than a fixed mass sample [13]. The ICE Futures Robusta Coffee Futures Contract, by contrast, evaluates a minimum 300 g laboratory sample and expresses defects and foreign matter as percentages by weight [14]. The exchange framework is itself in transition: ICE Futures U.S. is phasing out the Coffee “C” contract in favor of the Coffee “C” Metric contract (listing submitted 21 August 2025), whose rule filing carries the grading standard over unaltered, including the schedule of imperfections, the origin-specific bases, and the cup criteria [13,36].

### 3.2. National and Regional Systems

The national systems show the same non-equivalence. Brazil’s Ministry of Agriculture, Livestock and Food Supply (MAPA) Normative Instruction (IN) 8/2003 uses a 300 g working sample and defect equivalents, with a stated order for resolving multiple defects on an individual bean [15]. Colombia’s export resolution uses a 500 g defect sample and *faltas*, while Peru’s implementation guide applies a 300 g laboratory sample with ISO-oriented quality-impact units [16,17]. Mexico specifies a 300 g basis in its green coffee standard but combines defect limits with other specification criteria, while Costa Rica’s reference chart uses category-equivalent logic without a sample mass in the cited reference document [18,19].

El Salvador’s regulation works with 300 g or a volume basis in its documented materials, and Nicaragua specifies 300.0 g with fractional defect calculations that are not rounded [20,21]. The East African Community (EAC) standard uses black-bean equivalence factors and distinguishes Arabica and Canephora values, but its accessible historical text does not state a defect-count sample mass. A harmonized revision (Draft East African Standard, DEAS 130) has been in the EAC pipeline. In the authors’ verification of the EAC and national-bureau catalogs (August 2026), no adopted revision could be identified, so the 1999 edition is treated here as the operative text [22]. Uganda combines counts, percentages, and penalty or bonus points in official forms, while Tanzania uses a 300 g sample and point values with separate grade requirements [23,24].

Indonesia’s national standard (Standar Nasional Indonesia, SNI) assigns *nilai cacat* on a 300 g sample, Vietnam’s current system uses percentage by mass for defects and foreign matter, and Papua New Guinea uses defect equivalents per kilogram for several green coffee grades [25,26,27].

The scope follows the systems compared in the ICO harmonization of International Coffee Council (ICC) Resolution 420 with the ISO defect chart and in the ICO quality-improvement program, through which origins not treated individually here (for example Ethiopia, Kenya, India, Guatemala and Honduras) remain accessible [31,32]. Table 1 selects ten systems that illustrate the principal architectures. Black bean is used as the comparison column because it is the only defect defined in every system considered and is the unit defect in most of them (one full black bean equals one full defect, imperfection, falta, or point), so that differences in its treatment expose differences in the underlying architecture, not in defect definition. The black-bean entries reproduce the rules’ own definitions and do not constitute a cross-system conversion. “Not stated” means that the cited accessible document does not provide a comparable sample mass for that procedure, not that the system necessarily uses no sampling protocol.

### 3.3. Why Harmonization by Arithmetic Is Unsound

Three aspects prevent simple interchangeability. First, the sample unit differs: 350 g, 300 g, 500 g, per kilogram, and a volumetric tray describe different observation bases [2,3,13,16,27]. Second, the object being counted differs. A black bean can be a complete bean, a partly black bean, a percentage by mass, a coefficient-bearing visual category, or one element in a defect-priority rule [1,3,14,15]. Third, the decision rule differs: ISO is a reference framework, the SCAA/SCA handbook is a specialty grading guide, exchanges govern contract delivery, and national rules may combine legal, export, screen size, moisture, and cup requirements [1,3,13,14,15,16].

Consequently, a calculated conversion of 500 g Colombian *faltas* to a 300 g system would add numerical precision without preserving the original rule. The appropriate comparative conclusion is not that one system is more or less strict in the abstract; it is that each system measures a specific operational construct. This is central to aroma research because a category defined for commercial counting may group samples with heterogeneous causes and precursor profiles.

## 4. Defect Typology and Causal Pathways

### 4.1. A Stage-of-Origin Approach

Etiological grouping is more informative than a single visual list because the same appearance may be reached by more than one pathway and a single pathway may produce several appearances. For the present review, defects are grouped by the stage at which the relevant disturbance is initiated: pre-harvest physiology; harvest and cherry maturity; wet or dry processing; drying; storage and transport; insect or microbial attack; and dry milling or other mechanical handling. This organization does not replace the definitions in standards. It instead provides a hypothesis structure for deciding which chemical variables, microbiological tests, or sorting records are relevant to a given lot [1,2,37,38].

Pre-harvest physiological defects arise when fruit or bean development is incomplete or structurally abnormal. Sucrose metabolism changes during coffee fruit development, and maturity-stage studies show that fruit composition and sensory quality vary across developmental stages [39,40]. An immature bean is therefore plausibly associated with an early interruption of reserve deposition, whereas malformed forms and shells are primarily developmental configurations rather than established chemical defect classes [1,41]. A shriveled or emaciated bean may reflect impaired filling, but the exact compositional consequences of that morphology should not be assumed in the absence of direct analysis.

Harvest-related defects begin with the selection of cherries at heterogeneous maturity or with fruit falling, remaining on the ground, or being retained too long before processing. Defective bean studies associate black and sour groups with differences in sugars, lipids, CGAs, and other constituents, while standards use appearance-based categories that may aggregate several causal histories [1,42,43]. Overripe or damaged fruit can provide conditions for microbial and oxidative change, but a brown appearance alone does not identify the organism, duration, or reaction sequence responsible for the result.

Wet processing introduces mechanical, enzymatic, and microbial pathways. Depulping can cut or crush beans when machine settings, fruit properties, or foreign material create damaging forces; the visible pulper-cut category is accordingly a process indicator, not by itself proof of a fermented cup note [1,3]. Wet processing also transiently affects seed physiology and exposes material to water-mediated biochemical and microbial change. Experimental work shows reactivation of germination-related processes during post-harvest treatment, while microbiological and metabolomic work documents dynamic changes from cherries through wet processing [44,45,46]. Such changes can be desirable when managed; they become defect-relevant when timing, oxygen exposure, hygiene, or fermentation conditions produce unwanted trajectories [47,48,49].

Drying rate and method affect green bean composition and the aroma-active compounds subsequently observed after roasting [38,50,51]. Rewetting, interrupted drying, and uncontrolled temperature can alter moisture distribution, membrane integrity, and the conditions for microbial growth. Faded and blotchy/whitish categories are frequently attributed to drying or storage history, but their visual appearance does not establish a common volatile profile or cup effect [1,52,53].

Green coffee storage reduces seed viability and changes potential aroma-precursor pools, including free amino acids and sugars, while accelerated storage studies show lipid oxidation changes that depend on packaging conditions [53,54]. These mechanisms are pertinent to faded, old, or past-crop material, but “old” is a storage history and sensory description, not a single visual defect category in all standards. Storage also creates an ecological window for fungi; water availability and temperature influence fungal growth and ochratoxin A (OTA) production, requiring a distinction between odor, visible mold, and toxicological risk [55,56,57].

The coffee berry borer, *Hypothenemus hampei*, is a major pest, and infestation level is associated with changes in physical grading and chemical composition of damaged beans [58,59]. Entry holes, internal galleries (the tunnels excavated by the borer inside the bean), and material loss may additionally facilitate microbial colonization, but the hole count does not itself demonstrate fungal contamination or a particular cup flavor [60,61]. Standards also set different thresholds for slight and severe insect damage, so a category boundary is an institutional decision as well as a biological observation [3,21,25].

Broken beans can roast differently because their geometry and surface-to-volume ratio differ. A study in Canephora found adverse sensory effects at a defined proportion of broken material, but this does not establish a unique green-bean volatile marker [62]. Parchment, pods, and husks are residual fruit tissues that identify incomplete separation, whereas shells are developmental bean malformations that become visible at processing (Section 5.10), and stones and twigs are non-coffee foreign matter [1,3]. The principal visual classes discussed in this section and in Section 5 are illustrated in Figure 1.

### 4.2. Causal Ambiguity and Evidence Discipline

The etiological label attached to a defect should match the strength of available evidence. Field physiology, controlled processing experiments, and direct chemical comparisons can support a causal proposition; a standard’s descriptive annex, a handbook descriptor, or visual convention may instead provide a useful hypothesis. Black, sour/brown, and insect-damaged beans illustrate this difference. Their grouping in standards does not prove a single origin, but independent compositional and volatile studies show that these sample classes differ from sound coffee [4,5,42,43,59]. Conversely, dark-green, bleached, malformed, and pulper-cut categories have more limited defect-specific aroma chemistry, although they are relevant to grading [1,3].

A further limitation is mixture. Commercial defects are often collected from lots instead of being produced under one controlled pathway; black beans may include fruit affected by delayed processing, disease, prolonged ground contact, or other deterioration. This heterogeneity can inflate apparent differences while obscuring the mechanism of any individual bean. Studies that compare clearly selected classes remain useful for quality control and screening, but their markers should be interpreted as class-associated unless the study controls cause, origin, processing, storage, and roast [4,5,42,63]. The review therefore uses “associated with,” “reported in,” and “marker” where the evidence does not demonstrate an exclusive causal mechanism.

## 5. Chemical Composition and Aroma Precursors of Defective Beans

### 5.1. Precursor Logic and Analytical Limits

The green coffee bean contains carbohydrate, nitrogenous, phenolic, and lipid pools that shape roasted aroma through overlapping thermal reactions. Sucrose is an important contributor to the formation of alpha-dicarbonyl compounds during roasting, and roast degree changes the reaction routes that yield alpha-dicarbonyls and other aroma-relevant products [8,9]. Amino acids participate in Maillard and Strecker reactions; trigonelline, CGAs, and lipids follow additional thermal and oxidative pathways [7,10,11,64,65]. Defect chemistry is therefore best interpreted as a shifted precursor balance, not as a simple search for a one-compound explanation. The baseline against which defect-associated deviations must be judged is the key-odorant set of sound coffee, established for roasted Arabica through sensory studies of character-impact odorants and for raw Arabica through analyses of potent odorants and their changes during roasting, and compiled in monograph form [66,67,68]. Reviews linking quality grading to volatile chemistry and compiling candidate markers frame this field [69,70].

The analytical evidence has three recurring forms. First, compositional studies quantify non-volatile differences, such as sucrose, CGAs, caffeine, trigonelline, lipids, reducing sugars, or biogenic amines. Foundational comparisons reported differences among black, sour, immature, and sound beans; proximate composition and fatty-acid profiles were established for green and roasted defective beans; later work quantified amine profiles across several defective classes; and green bean chemistry has been correlated with cup quality in graded Brazilian lots [42,43,71,72,73]. Second, headspace or mass-spectrometric work distinguishes classes through volatile fingerprints or ionization patterns, identifying candidate markers in black, sour, immature, broken, and insect-damaged material [4,5,6,74]. Third, a smaller set of studies connects an individual off-flavor syndrome to a chemically defined compound, as with 2,4,6-trichloroanisole (TCA) in Rio flavor and 2-isopropyl-3-methoxypyrazine (IPMP) in potato taste defect [75,76,77,78].

These evidential forms should not be conflated. A volatile may be statistically discriminating without being odor-active; an odor-active compound can be highly potent without explaining all perceptual variation in a defect class; and a measured non-volatile difference may alter roasting potential without surviving into the beverage as a discrete aroma. GC-O links chromatographic separation to human odor perception, and odor activity values (OAVs) relate concentration to threshold, but neither method has been applied comprehensively across the visual defect classes used in trade [4,7,10]. The absence of a full chain is especially important when a handbook descriptor is repeated as if it were a confirmed molecular diagnosis. Odor thresholds are deliberately not tabulated in this review: they are strongly matrix-dependent (values determined in water, cellulose, coffee brew, and roasted coffee differ by orders of magnitude), so a single number per compound would misrepresent the underlying data; readers are referred to the key-odorant literature for threshold and odor-activity values [66,67]. Survival of green bean markers into the roasted product is likewise compound-specific and mostly unmeasured: most potent odorants of raw coffee do not persist through roasting [67], and among the compounds reviewed here, persistence is demonstrated only for the fermentation-associated esters [79], TCA [75], and IPMP [78].

### 5.2. Black Beans

Black beans are usually defined morphologically by a partially or completely black endosperm or surface, but that observation can integrate developmental damage, fermentation, decay, and drying history [1,3]. In compositional comparisons, black beans differ from sound beans in several non-volatile variables, including reported changes in sucrose, lipids, CGAs, trigonelline, and caffeine across studies and sample sets [42,43].

Volatile studies provide more specific, yet still mainly marker-level, evidence. Toci and Farah identified differences in the volatile composition of defective beans and reported marker candidates for black material; subsequent fingerprinting extended the set of low-quality indicators across defect categories [4,5]. Aldehydes and heterocyclic compounds reported in defective classes can reflect oxidation, microbial activity, or altered roasting substrates, but their presence in a headspace profile is not equivalent to proof that a particular molecule is responsible for a black-bean cup note.

More precisely, black beans can contribute to a less favorable precursor balance and uneven roast behavior; there is no universal “black” flavor.

### 5.3. Sour/Brown Beans

Sour or brown beans, including categories often termed *ardido*, combine visible brown or reddish-brown material with a defect concept frequently linked to fermentation or deterioration [1,3,15]. Compared with sound coffee, defective-bean studies report compositional differences that include changes in sugars and other chemical variables; volatile-marker studies identify sour-bean-associated signals such as acids, aldehydes, and phenolic candidates [4,5,42,43].

Organic acids influence sourness and overall flavor in coffee, but sensory effects depend on acid identity, concentration, matrix, and roast context [80]. Post-harvest microbial metabolism can consume or transform available substrates, while processing conditions shape the microbial and metabolomic trajectory from fruit to green coffee [38,46,47,48]. This provides a mechanistic basis for considering uncontrolled fermentation as one possible sour/brown pathway. It does not show that every brown bean arises through the same organisms or that every sample will produce the same fermented note.

Lower or shifted carbohydrate availability may affect Maillard balance, and microbial metabolites or oxidation products may persist, degrade, or react further [8,9,10,11]. In mixture testing, consumers detected sensory changes from a 10.25% share of black, green, and sour beans in healthy Arabica, but acceptance was not reduced and hedonic thresholds could not be estimated, so no universal concentration threshold for *ardido* can be derived [81]. The leading descriptors should therefore remain broad (sour, fermented, harsh, or astringent) unless a defined sensory study and material support a narrower claim.

### 5.4. Immature Beans

Immature beans arise from cherries harvested before completion of development, making maturity a central explanatory variable and more than a color category. Fruit-development work shows changes in sucrose metabolism during coffee development, and maturity-stage research reports differences in chemical composition and sensory quality across fruit stages [39,40]. Chemical comparisons of defective material have found immature beans to differ from sound beans in sucrose, CGAs, and related composition variables [42,43]. Amine profiles differ between unripe and sound beans [72] and are also processing-dependent within unripe material; however, that processing study contained no mature control (all treatments were unripe coffee), so it showed process dependence, while the unripe-versus-sound contrast rested on the direct comparison [72,82].

Sucrose accumulation and tissue development are incomplete relative to fully mature material, so the carbohydrate and nitrogen balance available for roasting differs [39,40]. This may change the formation of Maillard-derived products and help explain why immature material often develops differently under a common roast program [8,9,10]. It is not scientifically adequate to infer a fixed compound profile from the term “immature.”

Sensory references commonly associate immature coffee with green, grassy, cereal-like, hay-like, or astringent character, and research on maturity confirms that fruit stage can affect sensory quality [3,40]. The often repeated association with “peanut” needs more restraint. A traditional sensory label is not equivalent to an established raw-bean aroma or to a defect-specific pyrazine mechanism; available marker studies and the broader roasting literature do not support that simplification [4,7,10]. In practical terms, immature beans merit separation because they combine an identifiable harvesting pathway with altered precursors and uneven roasting potential, not because one descriptor can diagnose them.

### 5.5. Quakers: A Documented Definitional Conflict

The word quaker must be interpreted in its normative and differentiated senses side by side. The standards are uniform on this point: ISO 10470:2004 heads its clause 4.5 entry “Immature bean; ‘quaker’ bean”, treating the two as one visual category with sensorial concern, and the SCAA handbook (p. 18), like the current SCA defect guide (edition 3, p. 23), lists “Quaker (when roasted)” as an alternative name of the immature bean within the secondary-defect structure [1,3,83]. Under this uniform normative position, quaker is the roasted manifestation of an immaturity pathway, and the qualifier “when roasted” makes the roast-appearance basis of the term explicit. This terminology is consequential because it directs attention toward early harvest, incomplete maturation, and the precursor profile discussed above.

Coffee Consulate’s differentiated material instead distinguishes an emaciated, light, shriveled bean from the immature bean. In that differentiated position, quaker denotes a nutrient-deficiency or exhausted-bean etiology, while immature beans retain an early-harvest and ripening pathway [12]. Physiological research supports the general proposition that coffee seed development and fruit filling depend on developmental and source–sink processes, but the differentiated material does not justify assigning a universal measured sucrose, amino-acid, or volatile profile to all emaciated beans [39,40,84]. The chemical evidence for the differentiated class is therefore much weaker than for experimentally selected immature beans.

The disagreement should not be resolved by choosing one label and silently rewriting the other. For grading, the official standard or contract definition remains controlling. For research, the differentiation matters because visually similar light or wrinkled beans could derive from different causal pathways and hence carry different precursor profiles. A study that pools early-harvest immature beans with physiologically emaciated beans may dilute or misattribute chemical differences. Future work should recruit both classes independently, document morphology and density, establish field or harvest history, and compare their green composition, roast development, and GC-O profiles under the same protocol [1,3,7,12].

A related practical implication concerns roast color. The only quaker-specific detection study identified in this review operates on exactly this basis: terahertz transmission separates quakers defined operationally by post-roast pallor, in agreement with the “when roasted” qualifier of the SCA texts, but it measures a physical proxy (a moisture and density contrast), not a precursor; the measurement is thus performed on the green bean, the quaker label is assigned only after roasting, and the green-state quaker remains chemically unaddressed [85]. A light roast response in immature material is not proof that every shriveled bean behaves identically, just as a wrinkled surface is not sufficient evidence of the same physiology. The appropriate conclusion is a differential diagnosis: retain the standard’s immature/quaker category for its stated purpose, while recording the alternative emaciation hypothesis separately when the morphology and supply-chain history warrant it [1,3,12].

### 5.6. Fermented Beans and Stinkers

“Fermented” and “stinker” refer primarily to off-odor or cup categories and do not describe a single visible morphology. ISO 10470 includes stinker/fermented coffee under defects primarily identified during cupping, which appropriately signals that visual sorting cannot resolve every relevant quality problem [1]. Wet-processing studies show that microbial communities and metabolites change during conversion of cherries to coffee, and fermentation reviews describe yeast and other microbial roles in the formation of flavor-relevant compounds [46,47,48,49].

When fermentation is controlled, these transformations can support desired quality; the defect problem is loss of control, unsuitable timing, hygiene, or oxygen conditions. The chemical evidence for a universal stinker signature is nevertheless limited. Volatile markers found in defective groups can be useful for discrimination, but a marker list does not establish that every stinker has a shared aroma-active compound or that raw visual inspection will detect it [4,5]. Research should thus distinguish process metadata, microbial ecology, green-bean volatile profiling, and sensory confirmation instead of treating “fermented” as a molecular endpoint.

Defined mixture studies coupled to roasted GC-O and controlled cupping would be needed to establish dose–response relationships for stinker material [10,11,38].

### 5.7. Moldy and Earthy Material, Including OTA

Moldy or earthy material brings sensory and food-safety questions into the same lot, but they are not the same question. Filamentous fungi occur during coffee fermentation, drying, and storage, and fungal growth and OTA production are governed by environmental conditions including water activity and temperature [55,56,86]. The defect-class evidence has three distinct layers. Ochratoxigenic fungi occur at higher incidence in defective material, including borer-infested beans [61,87]. OTA itself has been measured in naturally defective graded fractions, with the highest concentrations in black (25.7 µg/kg) and sour (11.3 µg/kg) beans and low findings in immature beans [87], and it has been quantified in beans classified by borer-infestation level [61]. The quantitative formation data as a function of water activity and temperature derive, by contrast, from experimentally inoculated, sterilized beans [55,56]. These layers justify targeted monitoring and preventive sorting, consistent with the Codex code of practice, which counts the removal of defective beans among its preventive operations [88]; they do not justify the assumption that each visually moldy bean contains OTA or that OTA causes a moldy aroma.

OTA is a toxicologically relevant mycotoxin; it is not an aroma marker. Reviews on coffee contamination emphasize prevention across the production chain, while analytical and storage controls address the conditions that permit fungal growth and toxin formation [55,57,89]. This distinction is important for quality systems: a sensory panel may detect a moldy or earthy note without measuring OTA, and a toxin result cannot be inferred from odor alone. The regulatory architecture reinforces this separation. At the international level, the Codex Alimentarius has adopted a code of practice for the prevention and reduction of OTA contamination in coffee, whereas no Codex maximum level has been established for coffee [88]. As a regional example of binding limits, under Regulation (EU) 2023/915, the European Union sets maximum levels for OTA in roasted coffee (3 µg/kg) and soluble coffee (5 µg/kg), while no maximum level is defined for green coffee itself. The toxicological basis of that framework is the European Food Safety Authority (EFSA) risk assessment of ochratoxin A in food [90,91]. Corresponding national limits in other jurisdictions differ in value and product scope and are not reviewed here.

Fungal volatile compounds can influence sensory quality, and fungal studies have examined volatile production with possible implications for coffee beverage quality [92]. Geosmin is a well-established microbial metabolite associated with earthy–musty odors in foods and water; this provides a chemically plausible explanation for some earthy notes [93]. It should nevertheless be reported as a candidate mechanism only when it has been measured in the coffee material under study. Green coffee needs defect-specific GC-O, concentration, and sensory work before visible mold, geosmin, earthy perception, and OTA can be treated as one causal chain.

### 5.8. Faded, Whitish, and Aged Material

Faded, white, or blotchy categories commonly point to drying and storage history. Storage research documents loss of green coffee viability and changes in potential aroma precursors, while storage and packaging studies identify lipid oxidation changes and sensory-relevant deterioration [53,54,94]. Drying-temperature work further shows that drying conditions influence later storage quality [52].

The chemical signature is not identical for all pale beans. A uniformly faded bean, a blotchy/spotted bean, an amber bean, and a low-density floater are defined differently in standards and may have different histories [1,3,16,21]. Storage experiments under defined time, moisture, and temperature quantify the odorant side of this pathway: over nine months of warm storage at elevated water content, methyl 2-methylbutanoate rose from 9 to 500 µg/kg, methyl 3-methylbutanoate rose from 0.6 to 80 µg/kg, 2-methoxy-4-vinylphenol (4-vinylguaiacol) rose from 390 to 15,000 µg/kg, and 2-methoxy-5-vinylphenol appeared where it had not been detectable, while lowering temperature or water content suppressed these changes [95]. The seed-physiology side is covered by the viability and precursor-pool work cited above [53]. This evidence derives from storage experiments, not from visually graded “faded” beans; it characterizes the aging pathway, not the sorting class, and does not substitute for a defect-specific study of each visual class [53,95,96].

The relevant controls are storage duration, temperature, oxygen exposure, packaging, moisture, and water activity, with color used as one observation among several [52,53,54].

### 5.9. Insect-Damaged Beans

The chemical evidence for coffee berry borer damage is stronger than for many other visible categories because infestation can be graded and compared. Recent work reports that increasing infestation changes physical quality and chemical composition, while volatile profiling of damaged green beans identifies differences correlated with infestation severity [59,97]. The compositional changes are severity-dependent: only at the highest damage grade did leachate electrical conductivity, an indicator of cell-membrane damage, roughly double, while potassium leaching, acetic acid, and succinic acid increased, and total sugars and lipids decreased, with lower grades statistically indistinguishable from undamaged beans. In that study, “Clean” denotes beans with one to two orifices without internal discoloration, with the undamaged reference being the “Control” [59].

Hexanal and 2-pentylfuran rise with the proportion of damaged beans, not with the mere presence of damage: in a blend-proportion design (percentages denote the share of damaged beans in a blend, not severity grades), hexanal was unchanged at a 20% share of slightly damaged beans and increased only at high shares and in heavily damaged material [97]. The fatty-acid profile of bored beans is nevertheless unchanged even where bulk composition differs [71]. The sensory role of these volatiles in a roasted beverage remains to be established for the individual damage class [97]. More severe infestation may differ from slight damage not simply by hole number but by gallery development, endosperm loss, and likelihood of secondary colonization. This is why standards’ numerical thresholds should not be mistaken for biochemical thresholds [3,21,25].

Fungi associated with borer galleries and the association between infestation, fungal contamination, and OTA risk make it reasonable to investigate damaged beans for mycological hazards [60,61,87]. Yet neither an insect hole nor a volatile marker proves a toxin concentration, and a sensory complaint cannot identify a particular microbial species. Integrated studies that pair damage severity, mycobiota, green chemistry, roasted aroma, and cupping are still needed.

### 5.10. Broken Beans, Shells, Parchment, and Husk

Physical fragments and residual tissues are frequently important to grading and roasting even when their defect-specific chemistry is sparse. Broken beans result from mechanical fracture, often in relation to moisture condition and milling forces; altered size can produce faster or less uniform heat transfer during roasting [62,98,99]. A Canephora study found that a defined proportion of broken beans affected sensory quality, which supports a practical roast-consistency concern but not the assignment of a universal molecular marker [62].

Shells are developmental structures exposed by processing, while parchment and husk are different residual fruit tissues that indicate incomplete separation [1,3,100]. Their major effect may be physical (uneven roasting, charring, contamination of the lot, or grade non-conformity) rather than a known green-bean precursor shift. Volatile-marker work has now reached these classes directly. Profiling of roasted Canephora across twelve defect categories, including broken, floater/spongy, shell, parchment, husk, and foreign matter, for which no defect chemistry had previously been reported, tentatively identified 42 candidate compounds. It separated bean from non-bean defects as the primary multivariate axis (57.8% of the variance on the first two components; three clusters), and selected candidates remained detectable in commercial-grade samples from three origins [101]. The identifications are, by the authors’ own statement, tentative (no authentic standards, no Kováts confirmation), and the sensory bridge remains incomplete [4,5,101]. It is consequently preferable to say that these materials can impair lot uniformity and may contribute undesirable effects at sufficient inclusion rates than to assign them a definitive standalone cup aroma.

Pods and husks also deserve a mycotoxin distinction because plant residues may carry different microbial exposure histories. The fact that coffee by-products have distinct compositions does not establish their behavior when accidentally included in green coffee, nor does it make a pod or husk a direct OTA marker [57,100,102].

Table 2 summarizes the current evidence without implying that all listed markers are odor-active in the cup. No grading of volatile flavor intensity is undertaken in this review: volatile findings are reported as class markers (statistical discrimination between sample groups), as measured concentrations where the source quantified them, and as odor-relevant only where the cited source established this by GC-O, OAVs, or sensory testing; the evidence tags in Table 2 record this status claim by claim. Reported thresholds and OAVs are population-level central tendencies; olfactory thresholds vary substantially both between individuals and within the same individual over time [103]. This variability is a further reason why fixed thresholds are not tabulated here and why sensory confirmation requires panel-based testing instead of single-assessor impressions. “Marker” includes non-volatile compositional shifts and class-discriminating volatiles; descriptors are leading sensory associations, not universal diagnoses.

## 6. Special Off-Flavor Syndromes

### 6.1. Rio Flavor and 2,4,6-Trichloroanisole

Rio flavor is a useful counterexample to the assumption that all meaningful green coffee defects are visually sortable. It is classically described as a musty, medicinal, or iodine-like off-flavor, and analytical investigations identify 2,4,6-trichloroanisole (TCA) as the compound responsible for the Rio character in green coffee, quantified at 15 to 106 ppb in affected green lots and accompanied by 2,4,6-trichlorophenol at 7 to 42 ppb [75]. More recent work developed rapid chemical-ionization mass-spectrometric screening for TCA in green *Coffea arabica* from different origins [76].

The syndrome does not, however, convert Rio into a visual defect category. ISO 10470 provides a category for other off-flavors and separately includes stinker/fermented coffee, but it does not make Rio a visual black-, brown-, or moldy-bean type [1]. Nor do the SCAA/SCA handbook’s visual groups establish TCA as the mechanism of fungus-damaged, sour, or foreign-odor material [3]. In practice, Rio may occur alongside a lot’s visible defects or arise in a lot that appears visually acceptable; sensory and chemical screening therefore complement each other, and neither can replace the other.

A TCA result invites investigation of chlorophenol-related contamination pathways and materials, not generic removal of every dark or discolored bean. Conversely, a lot described as musty should not be labeled Rio without appropriate sensory and analytical evidence. The strength of the Rio literature lies in the explicit identification of a compound and its off-flavor relevance; this evidential standard should not be borrowed by analogy for less well-characterized categories [75,76].

### 6.2. Potato Taste Defect and IPMP

Potato taste defect (PTD) is another syndrome that may not have a uniform visible counterpart in green coffee. Studies of East African coffee identify 2-isopropyl-3-methoxypyrazine (IPMP) as a compound linked to PTD and to injury involving *Antestiopsis* bugs [77]. Quantitative analysis in roasted coffee has further supported IPMP as a severity marker, so that the chemical signal can be related to the intensity of the PTD syndrome [78]. In green coffee, IPMP behaves as an interior signal: whole-bean headspace sampling can return a false negative, so ground-bean sampling is required before absence is claimed [77]. The syndrome’s chemistry was first described in Central African coffee as a “peasy” note attributed to IPMP; the concentration scale in that early conference report differs by orders of magnitude from later determinations, so it is cited here only as a historical record [105].

The causal story remains more complex than a single pest label. The bacterium *Pantoea coffeiphila* has been described as the causal organism of the potato taste of Arabica coffee from the African Great Lakes region, with berry wounding facilitating its entry, which positions the bug as a vector or enabler, not a strict requirement [106]. Fungal taxa have been associated with PTD in Rwandan coffee [107], and experimental work reports that mechanical damage alone can produce PTD without *Antestiopsis* infestation, raising IPMP from 0.39 to 13.34 µg/g roasted coffee, a factor of about 34, in mechanically damaged berries [108]. The same experiment carries an instructive internal discrepancy. The caged-insect arm produced the most PTD-positive samples (three of four, compared with two of four for mechanical damage and none for the control). Its IPMP value, however, was intermediate between the other two arms and is reported only graphically. The chemical and the sensory ranking of the treatments therefore do not coincide, even though the authors report a positive logistic relationship between IPMP concentration and PTD detection overall [108]. Thus, insect injury, tissue damage, and microbial association should be considered interacting or alternative contexts; they do not form a closed universal chain. The relevant physical defect categories in standards, such as insect-damaged beans, slightly or severely damaged beans, or off-flavor coffee, can flag material for investigation, but they do not define PTD or measure IPMP [1,3,21,22,25].

PTD also cautions against the casual use of “potato” as a generic earthy descriptor. If the syndrome is being claimed, analytical measurement of IPMP and a suitable sensory protocol are preferable. If an earthy note is observed without such evidence, it should remain an earthy or musty sensory observation, potentially linked to several sources. This distinction preserves the value of both screening and cupping without treating one as proof of the other [77,78,107,109].

### 6.3. Geosmin and General Earthiness

Geosmin is widely recognized as a microbial metabolite responsible for earthy–musty odors in foods and water [93]. Coffee-specific measurements do exist: geosmin has been detected in green Rio-affected coffee and quantified at approximately 1 ppb in a single moldy/earthy green lot [75,104]. Such measurements are isolated and cannot be generalized, and it still does not follow that geosmin is the chemical basis of every earthy coffee or that an earthiness descriptor identifies its source; measurement in the material under study, ideally combined with odor activity and sensory data, remains necessary.

Standards generally treat these observations indirectly. ISO 10470 distinguishes visible mold-related or visual categories from off-taste coffee, while the SCAA/SCA handbook classifies fungus-damaged beans and recognizes foreign odor in grading practice [1,3]. Neither structure provides a chemical assay for geosmin, IPMP, or TCA.

## 7. Discussion

### 7.1. Defects as Precursor Shifts, Not Fixed Flavors

The principal synthesis of this review is that green coffee defects are best treated as deviations in material condition and precursor state (Figure 2). For black, sour/brown, immature, insect-damaged, and stored beans, the literature documents compositional or volatile differences relative to sound coffee [4,5,42,43,53,59]. The most plausible route to aroma relevance is then through changed roasting chemistry: altered sucrose and amino-acid availability, phenolic composition, lipid oxidation, pre-existing microbial metabolites, or physical heat-transfer differences influence the volatile mixture formed in the roaster [7,8,9,10,11,65].

A defective bean enters a blend at a certain fraction, has a specific moisture and density, follows a particular roast profile, and is diluted by sound material. A class marker therefore does not by itself set the concentration of an odor-active compound in the brewed beverage (Section 5.1), whereas a potent contaminant such as TCA can produce a notable syndrome despite the absence of a distinctive visual bean category [75,76].

This distinction is intended to refine commercial grading. Visual sorting is highly effective for objects with clear morphology, such as stones, pods, fragments, shells, and heavily bored beans, and it can remove material associated with increased quality or safety risk [1,3,110]. It cannot resolve every off-flavor syndrome, distinguish all causal pathways within a color group, or establish chemical safety.

### 7.2. Classification–Chemistry Mismatch

This review identifies a persistent mismatch between standard categories and the granularity of chemical evidence. Standards must use definitions that are repeatable in routine inspection, often based on visual presentation or simple counts. Chemistry instead reveals overlapping causes and transitions. The black, sour/brown, and insect-damaged classes illustrate this: their appearances can arise through several pre- and post-harvest routes, and category thresholds need not coincide with biological or chemical transitions, as discussed in Section 4.2, Section 5.2, Section 5.3 and Section 5.9.

The quaker controversy is a more fundamental mismatch because it concerns the object being studied: the two documented definitions (Section 5.5) select different materials, so reported precursor differences can be compared only when the operational definition is stated, the morphology is documented and the selection basis is recorded [1,3,12].

Non-interchangeability across countries and markets (Section 3.3) has a direct sampling consequence for aroma research: because a defect total carries no stable chemical interpretation across the differing bases, a study designed for aroma chemistry may need a defect-specific sample preparation finer than the commercial grade to avoid mixing several causes in one group.

The controlled-fraction evidence available across this review reduces to four studies of three kinds. Two are sensory mixture studies. In Canephora, cupping attributes varied with the broken-bean share and the roast level, with the best result at a 10% broken share under light roasting [62]. In Arabica, beverages containing 2% to 100% black, green, and sour beans among healthy beans were tested with hedonic-threshold methodology and check-all-that-apply profiling; consumers detected sensory changes from a 10.25% defective share, but acceptance was not reduced and hedonic thresholds could not be estimated [81]. One study is a chemical blend-share design without sensory testing: headspace volatile markers rose with the share of borer-damaged beans and were unchanged at a 20% share [97]. One study is a defect-induction field experiment: potato-taste cup incidence and the IPMP concentration ranked the damage treatments differently [108]. Across all four studies, no beverage-level sensory dose–response threshold for any visual defect class was identified within the search scope of this review.

### 7.3. Research Priorities

The priorities below are ordered from short-term needs, which are addressable with existing methods, to longer-term directions, which depend on the data that the short-term studies would generate. The most urgent short-term need is defect-specific sensory chemistry after roasting. Many studies establish differences in green bean composition or volatile fingerprints, but few close the chain with roasted GC-O, odor activity values, recombination or omission experiments, and blinded sensory work at known defect fractions [4,5,6,7]. Such studies should analyze green and roasted material from the same defined defects, including controls matched by species, origin, processing, crop age, and moisture. A fully standardized reference matrix for threshold determination is unlikely to be achievable for a natural product: the sound background itself varies with species, origin, and roast degree, and a stored reference lot would change during storage (Section 5.8). What can be standardized is the preparation and testing protocol. Future threshold, OAV, and recombination studies on defect compounds should therefore prepare samples according to ISO 6668 (light roast for defect assessment, medium roast for flavor assessment) [111], report the base-coffee characteristics, and determine odor activity in the coffee beverage itself, not in water.

A second short-term priority is causal sampling. Black and sour/brown beans should be collected from known histories, or generated through controlled pathways where ethical and practical, to separate delayed processing, microbial decay, heat damage, and other routes. Immature and emaciated/quaker material should be deliberately separated according to the two documented definitions before comparing sugars, CGAs, amino acids, trigonelline, lipids, biogenic amines, and roast development [1,12,42,43,72,82]. This is essential if the vocabulary controversy is to be resolved with data.

A third priority spans both horizons and concerns broader species coverage. Much of the defect-specific chemistry has focused on *Coffea arabica*, whereas Canephora is a major commercial species and is subject to classification systems with distinct grade rules [14,22,25,27]. The twelve-category volatile profiling of roasted Canephora integrated in Section 5.10 opens this direction but leaves the decisive steps unresolved: validation of the tentative candidates against authentic standards, odor-activity testing, and sensory confirmation are still required before any of them can carry a defect claim, and extension to green material and to further origins and harvests remains open [101].

These priorities also define the realistic role of artificial intelligence (AI) in grading, on the same two time horizons. The most immediate application is the grading procedure itself. Manually spreading and counting the defects in a 300 g, 350 g, or 500 g working sample is laborious and grader-dependent, and machine-vision detection and counting of defective beans is already being implemented. Examples range from deep-learning detectors that reach high accuracy on SCA-compliant defect categories at edge-compatible latency [112] to a mechatronic system that combines dual-sided whole-bean imaging of a standard 350 g sample, a public twelve-class defect dataset, and embedded real-time counting on low-cost hardware [113]. The latter processes a sample faster than an experienced grader, and such systems could plausibly also improve reproducibility. What still requires validation is the application of each classification system’s own counting rules, so that automation preserves the source-specific architectures (Section 3.3). Beyond counting, machine learning systems can already classify beans from images or spectra, and the terahertz example in Section 5.5 shows that such detection can operationalize a defect definition. For a flavor-integrated grading standard, however, the limiting factor is the labeled ground truth, not the algorithm. The corresponding longer-term direction is a staged, AI-assisted pathway toward such a standard. First, multimodal datasets must pair bean-level images or spectra with the stated operational defect definition, green and roasted composition, and panel-based sensory outcomes at defined defect fractions prepared according to ISO 6668 [111]. Models can then be trained to predict cup-relevant outcomes, not visual labels alone. Only then can candidate grading thresholds be derived and validated across the existing classification architectures without substituting for them. This direction is compatible with current institutional development, in which the SCA Coffee Value Assessment already combines physical with descriptive and affective assessment [30,33,34,35]. An AI layer would add the predictive link between bean-level measurement and cup outcome that the present evidence base does not yet support.

Finally, as a design principle across both horizons, food-safety and sensory research should remain linked but separate. Work on fungal growth and OTA needs appropriate water-activity, storage, and toxin measurement designs, while moldy or earthy flavor research requires volatile and sensory methods [55,56,57,87,92]. The same principle applies to insect damage and PTD: the borer, fungal associations, IPMP, and cup outcome are related research questions, not synonymous variables [61,77,78,107].

### 7.4. Implications for Grading and Roasting

Graders should record the governing standard, original sample basis, defect definition, count or mass measure, and whether the finding is visual, olfactory, or cup-derived. Do not convert a mass percentage to full equivalents or a 500 g *falta* count to a 300 g type as if this creates an equivalent result [14,15,16,26]. Where a lot raises an off-flavor concern without a clear visible category, retain the sensory observation and investigate it analytically where warranted.

For roasters, the relevant question is twofold: whether a lot meets a defect limit, and whether the defect composition predicts uneven thermal behavior or changed precursor availability. Broken or small material can roast at a different rate, immature material may display altered development, and stored material may have lost some flavor potential [40,53,62]. A single roast profile cannot be expected to restore depleted precursors or neutralize every contaminant; sorting, blend design, and storage management therefore remain upstream quality tools.

For laboratories, a tiered strategy is appropriate. Routine physical sorting and moisture checks (the loss-in-mass method was revised as ISO 6673:2025) address visible and process-related defects; targeted chromatography can investigate suspect volatile syndromes such as TCA or IPMP; toxin assays address OTA independently; and sensory protocols test whether a measured difference has cup relevance [2,29,57,76,78].

### 7.5. Limitations of the Evidence Base

The evidence compiled for this review has quantifiable concentration limits that should temper every marker statement made above. First, a single source dominates the volatile evidence: among the primary studies reporting compound-level comparisons for defective beans, one study provides by far the most extensive compound inventory across defect classes and thereby the clear majority of the compound-class associations drawn on above; its tables report directional differences without significance tests, and its own authors ultimately endorse only 2-butyl-3,5-dimethylpyrazine, at dark roast, as a low-quality indicator [5]. Any statement of the form “N compounds are associated with defect X” inherits that concentration.

Second, several graded classes have almost no measured chemistry. Broken/chipped/cut beans, floaters, withered beans, and foreign matter are fully defined, weighted, and counted in the trade standards. For broken, floater/spongy, shell, parchment, husk, and foreign matter, within the search scope of this review, the first volatile data as sorted classes appeared only in roasted Robusta and only at tentative identification level, no measurements of withered beans were identified, and quakers carry definitions and a physical detection proxy but no defect-specific chemical evidence [1,3,83,85,101]. For these classes, the grading label remains very nearly the entire evidence base. The normative comparison is itself limited in coverage: Ethiopia, Kenya, India, Guatemala, and Honduras are represented only through the ICO compilations, not by their own instruments [31,32].

Third, the OTA evidence for visually graded classes is thin and layered. One survey ties positive OTA determinations to naturally defective graded fractions, highest in black and sour beans and low in immature beans [87], and OTA has been quantified in beans classified by borer-infestation level [61]. Beyond these, the quantitative data derive from controlled inoculation of sterilized, rewetted beans, which establishes formation conditions rather than the toxin content of graded lots [55], and the one direct measurement on a moldy/earthy graded lot was negative [104]. Class-to-OTA claims therefore rest on few natural-contamination measurements and otherwise remain risk-based.

Finally, roast defects constitute a separate defect axis that must not be conflated with green bean classes. Marker compounds have been assigned to light, scorched, dark, baked, and underdeveloped roast faults produced from sound green coffee, and one of them, phenol, is also a recurring cup descriptor of several green defect classes; a phenolic or smoky note is not by itself diagnostic between a green bean defect and a roasting fault [114].

In addition to these evidence-level limitations, the review method itself is a source of potential bias. This article is a structured narrative review (Section 2); the literature was identified through keyword searches, citation chasing, and direct retrieval of normative texts, not through a fully systematic search and screening protocol, and no formal assessment of study quality or risk of bias was performed. Literature omission and selection bias cannot be excluded. The source hierarchy and exclusion rules stated in Section 2, the per-claim evidence tags in Table 2, and the single-source concentration disclosed above are intended to make the resulting selectivity visible; they cannot eliminate it.

## 8. Conclusions

Green coffee defect systems are indispensable for inspection and trade, but they do not constitute a universal chemistry or sensory scale. ISO, specialty, exchange, and national systems differ in sample basis, defect logic, and purpose; their counts and percentages must therefore be retained in their original form rather than converted across systems [1,2,3,13,14,15,16].

A precursor-centered interpretation provides a stronger bridge to aroma research. Black, sour/brown, immature, insect-damaged, and aged beans have documented compositional or volatile deviations, while roasting transforms those deviations through overlapping Maillard, Strecker, lipid, and other pathways [4,5,8,10,42,43,53,59]. For many physical or visual categories, however, the evidence remains primarily morphological or operational, and defect-specific odor-active evidence is missing.

The quaker terminology must remain explicitly dual. ISO/SCAA usage associates quakers with immature beans, whereas the differentiated Coffee Consulate position treats shriveled, emaciated beans as a distinct etiological class [1,3,12]. Scientific comparison requires both positions to be recorded, not harmonized by assertion. Rio flavor and PTD show how much stronger a defect account becomes when sensory syndromes are connected to defined markers such as TCA and IPMP [75,78]. Future studies should apply that standard (controlled causal sampling, matched roasting, GC-O or equivalent odor-active analysis, defined defect fractions, and blinded sensory testing) to the visual defects that currently dominate green coffee classification.

## Figures and Tables

**Figure 1:**
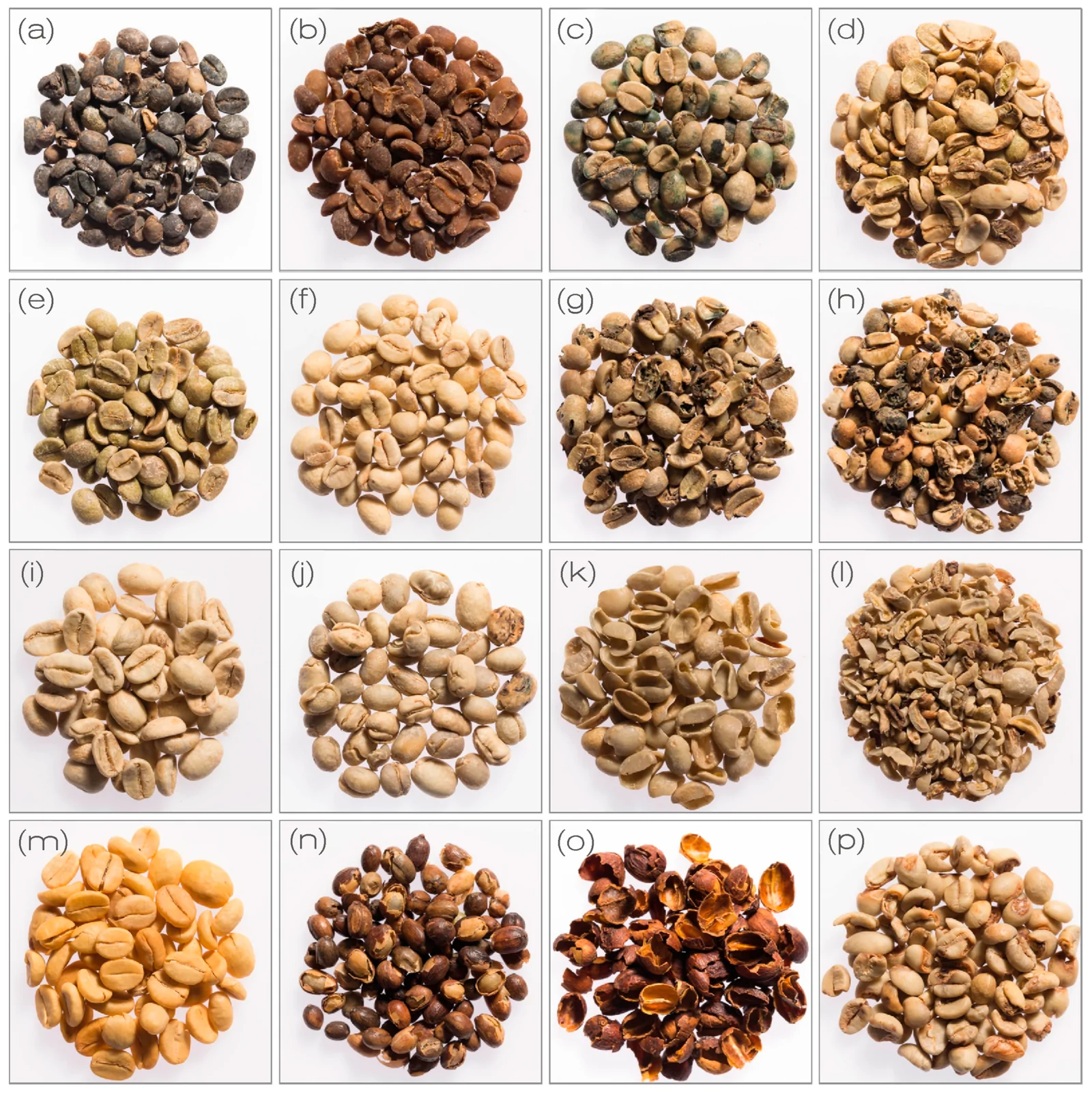
Principal visual defect classes of green coffee as discussed in Section 4 and Section 5, shown with the material of the EM0011 defect sample set [12]: (**a**) black beans; (**b**) sour/brown beans (*ardido*); (**c**) dark green beans; (**d**) underdeveloped, emaciated beans (the class the differentiated position terms quaker); (**e**) unripe beans; (**f**) faded beans; (**g**) eaten beans (slight insect damage); (**h**) well-eaten beans (severe insect damage); (**i**) bleached beans; (**j**) malformed beans (*madres*); (**k**) shells (*conchas*); (**l**) broken beans; (**m**) parchment coffee; (**n**) dried cherries (pods); (**o**) husk fragments (*cascara*); (**p**) pulper-cut beans. Photographs © Luca Siermann (fotodesign-siermann.de) for Coffee Consulate; reproduced with permission of the copyright holder.

**Figure 2:**
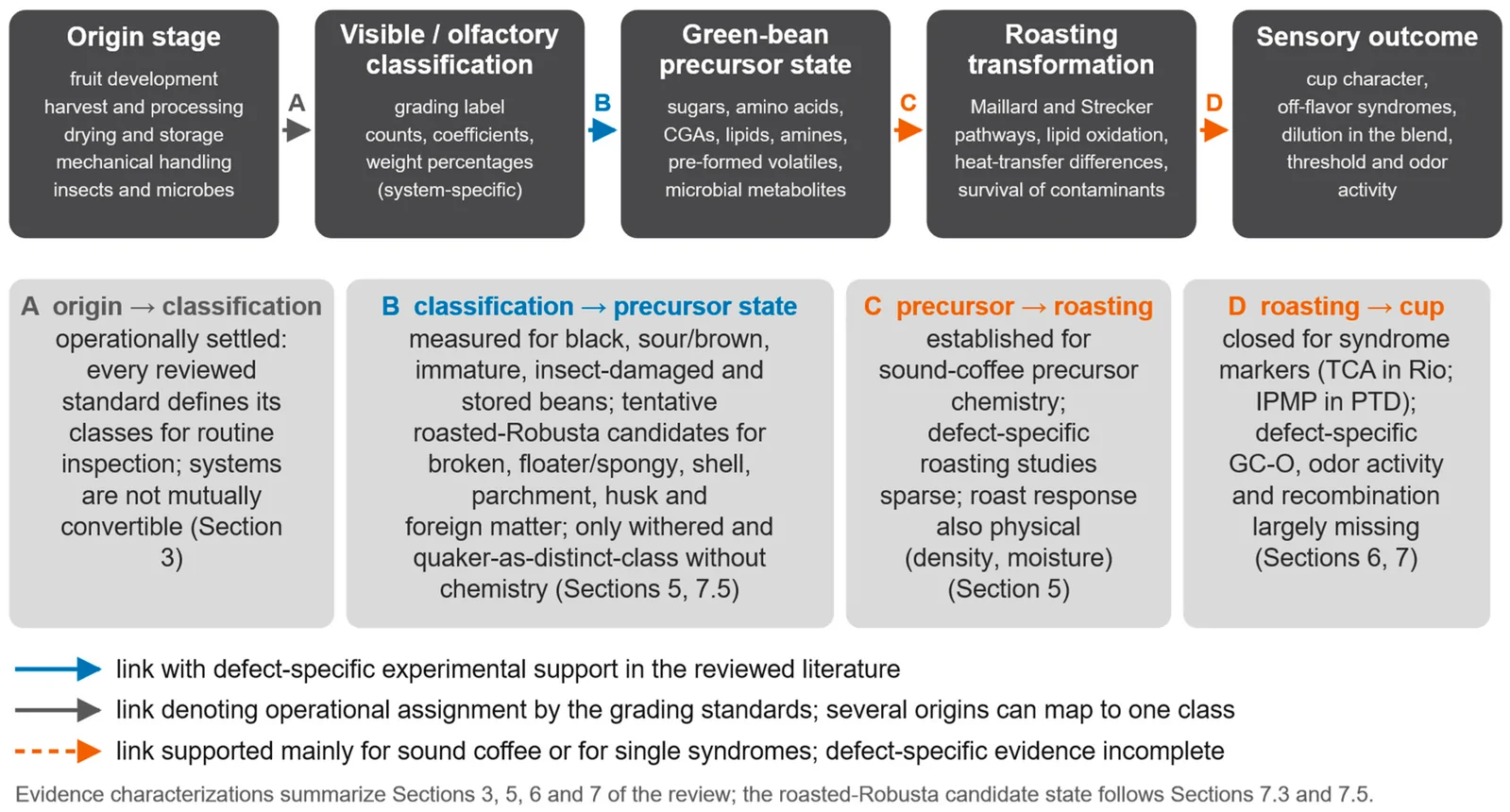
Causal chain linking defect origin to sensory outcome, with the level of defect-specific evidence available at each link. Upper boxes: the five stages distinguished throughout this review; lower boxes: the state of evidence for links A–D, with the sections in which it is discussed. The grey solid arrow at link A denotes operational assignment rather than experimentally demonstrated etiology: the grading standards define which visible class material is assigned to, and several origins can produce the same visible class (Section 3 and Section 4.2). The blue solid arrow at link B denotes defect-specific experimental support; orange dashed arrows denote links supported mainly for sound coffee or for single syndromes (TCA, 2,4,6-trichloroanisole; IPMP, 2-isopropyl-3-methoxypyrazine).

**Table 1:** Comparative architecture of ten green coffee defect classification systems; sample bases, counting logic, and black-bean treatment are reproduced as stated in the governing documents. The ten systems were selected so that each counting architecture occurring in the reviewed set (coefficients, defect equivalents with their priority-sequence and fractional variants, *faltas*, point values, summed *nilai cacat*, weight percentages, and volumetric imperfection counting) appears at least once. The systems not shown (Mexico, Costa Rica, El Salvador, East Africa, Uganda, Vietnam, and Papua New Guinea) each follow one of these architectures and are described in Section 3.1 and Section 3.2.

Standard or System	Sample Mass or Basis, as Stated	Counting Basis	Treatment of Black Bean, as Stated
ISO 10470:2004 with ISO 4149:2025 examination	300 g laboratory sample for ISO 4149 examination	ISO 10470 coefficients for loss of mass and sensorial concern; defects may be expressed by mass fraction	Black and partly black: 0 loss-of-mass and 1 sensorial-concern coefficient [1,2]
SCAA/SCA Green Arabica Coffee Defect Handbook	350 g green; 100 g roasted	Full defect equivalents; primary and secondary categories	Full black 1 bean = 1 primary defect; partial black 3 beans = 1 secondary defect [3]
ICE Futures U.S. Coffee “C” Rules	Volumetric tray	Full imperfections relative to an origin-specific basis	One full black bean = one full imperfection [13,36]
ICE Futures Robusta Coffee Futures Contract	300 g minimum laboratory sample	Percentage of defects and foreign matter by weight	Black bean is defined by extensive external surface and endosperm blackening within the weight-percentage method [14]
Brazil MAPA IN 8/2003	300 g working sample	Defect equivalents with a multiple-defect priority sequence	One *grão preto* = one defect [15]
Colombia Resolución 02/2016	500 g for defects	*Faltas* in two groups	One full black bean = one *falta*; two partly black beans = one *falta* [16]
Peru NTP/GIP implementation	300 g laboratory sample	ISO-oriented quality-impact units and equivalents	One black bean is listed as one equivalent in the stated annex logic [17]
Nicaragua NTON 03 025-11	300.0 g	Defect equivalents including fractional results	1 black bean (grano negro) = 1 defect; 3 partial black beans (grano negro parcial) = 1 defect (1 bean = 0.33); defect sums are not rounded [21]
Tanzania Coffee Industry Regulations	300 g processed coffee	Defective-points count with individual and fractional point values	Black = 1 point; half black = 1/2 point [24]
Indonesia SNI 01-2907-2008	300 g	Summed *nilai cacat*	1 black bean (biji hitam) = 1; 1 partly black bean (biji hitam sebagian) = 1/2; 1 broken black bean (biji hitam pecah) = 1/2 [25]

**Table 2:** Reported chemical markers, precursor shifts, and leading sensory descriptors for major green coffee defect classes and off-flavor syndromes; markers include non-volatile compositional shifts and class-discriminating volatiles without implied odor activity. Tags in parentheses give the matrix in upper case (G = measured in green beans, R = measured in roasted material), followed after a slash by lower-case letters on four independent dimensions: statistical reporting (q = quantified with inferential statistics reported in the source; d = directional comparison without significance testing), replication (s = single lot or single study), identification confidence (t = identification reported as tentative by the source, without authentic standards or retention-index confirmation), and sensory validation (v = direct odor-activity or sensory evidence in the cited source, such as GC-O, an aroma value or cup testing); publication type is marked separately (c = conference literature). Letters from different dimensions combine independently, e.g., (G/q,s); the absence of a letter means that the dimension does not apply or is not uniformly reported across the sources of that claim. Control matching and sample definitions that affect interpretation are stated in words within the cells. These tags describe what each cited source reports; they are not a formal quality or risk-of-bias assessment (Section 7.5). Descriptors derive from grading standards and defect-class literature, not from controlled recombination or omission panels. Odor thresholds are not tabulated because they are matrix-dependent (Section 5.1).

Defect or Syndrome	Key Chemical Markers or Precursor Shifts	Leading Sensory Descriptors and Evidential Qualification
Black beans	Reported differences in sucrose, lipids, CGAs, trigonelline, and caffeine (G), with directions varying between studies and matrices; class-associated volatile markers including oxidation- and heterocycle-related signals (G/R/d); electrospray ionization–mass spectrometry (ESI-MS) fingerprints separate defective from non-defective beans (G)	Fermented, dirty, moldy, or phenolic are reported descriptors; defect-specific odor-active confirmation remains limited [4,5,42,43,72,74]
Sour/brown (*ardido*) beans	Altered sugars and titratable acidity (G); candidate acid and aldehyde volatile markers (G/d); phenolic volatiles reported in roasted material (R/d)	Sour, fermented, harsh, or astringent; no single acid or marker proves the cup effect [4,5,42,43,72,80]
Immature beans	Differences in sucrose and maturity-related composition (G); altered amine profiles relative to sound beans (G); amine profiles additionally processing-dependent within unripe material, shown without a mature control	Green, grassy, cereal-like, hay-like, and astringent; “peanut” is not a confirmed defect-specific molecular diagnosis [3,39,40,42,43,72,82]
Quaker, differentiated emaciation position	Direct defect-specific precursor profile not established; no reviewed study measures quakers separately from immature beans; terahertz transmission, measured on green beans, provides a physical proxy (moisture/density) for a quaker status assigned after roasting by pallor (G, measurement stage; classification stage post-roast)	Standards are uniform (ISO 10470 clause 4.5 “Immature bean; ‘quaker’ bean”; SCAA handbook p. 18 and SCA defect guide ed. 3 p. 23 “Quaker (when roasted)”); the emaciation position is the differentiated physiological reading of the present author’s organization [1,3,12,83,84,85]
Fermented/stinker coffee	Ethyl 2-methylbutanoate, ethyl 3-methylbutanoate, and cyclohexanecarboxylic acid ethyl ester (CHEE) elevated in over-fermented green beans and persisting into roast and brew (G/R); CHEE the only candidate with an aroma value above 1 (10–20), but at sound-control levels in a Robusta sample, so universality fails (conference literature) (c,s,v)	Fermented or unpleasant off-odor; sensory outcome requires direct evaluation [1,46,47,48,49,79]
Moldy/earthy coffee	Geosmin (≈1000 ppt), 2-methylisoborneol (≈100 ppt), and 2,4,6-TCA (≈300 ppt) quantified in a single moldy/earthy green lot (s,v), located by GC-O after instrumental profiling alone failed to separate the samples (G); OTA negative in the same material; fungal growth and OTA formation governed by water activity and temperature	Moldy, musty, or earthy; visible mold, OTA and odor must be assessed separately [55,56,57,86,87,92,93,104]
Faded/aged material	Storage-based evidence (defined time, moisture, temperature; not visually graded “faded” beans): methyl 2-/3-methylbutanoate and 2-methoxy-4-vinylphenol strongly increased and 2-methoxy-5-vinylphenol newly appeared after nine months of warm, moist storage (G/q,s,v); reduced viability and changed precursor pools (G); lipid oxidation under storage (G)	Flat, stale, woody, or cardboard-like loss profile; visual pallor alone is non-specific [52,53,54,94,95]
Insect-damaged beans	Severity- and share-dependent changes (G): conductivity, potassium leaching, and acetic/succinic acid increased and sugars and lipids decreased only at the highest damage grade (q) (the study’s “Clean” = 1–2 orifices without discoloration; undamaged reference = “Control”); hexanal and 2-pentylfuran rose with the blend share of damaged beans, unchanged at 20% (G); fatty-acid profile of bored beans unchanged (G/R); alkanes and interior IPMP in *Antestiopsis*-damaged beans (G)	Bitter, astringent, sour, or diminished aroma are possible, but defect-specific roasted odor-active evidence is incomplete [59,60,71,77,97]
Broken beans, shells, and residual tissues	Primarily geometry, moisture/milling, and material-composition issues; no established defect chemistry; first tentative volatile candidates for broken, shell, parchment, and husk reported in roasted Robusta (R/d,s,t)	Uneven roasting or loss of uniformity; direct sensory effect depends on proportion and roast context [1,62,98,99,100,101]

## Data Availability

No new data were created or analyzed in this study. Data sharing is not applicable to this article.

## Appendix A

**Table A1:** Search and source-selection record of this structured narrative review.

Item	Record
Review type	Structured narrative review (see Section 2). This record documents the search and selection process at the level at which it was conducted; it is not a systematic review protocol.
Platforms and document sources searched	Bibliographic databases and publisher platforms: PubMed/PMC, ScienceDirect, Wiley Online Library, SpringerLink, ACS Publications, Taylor & Francis Online, MDPI, and SciELO. Standards and institutional sources: ISO Online Browsing Platform, national standards portals, and the official document servers of the ICO, the SCA, and ICE.
Keywords and mode of application	The keyword set listed in Section 2 was used singly and in combination across the platforms listed above, without field restriction and without a start-date restriction. Saved per-database search strings were not recorded.
Coverage period and language	Publications up to August 2026; no language restriction (sources in English, Portuguese, Spanish, German, Indonesian, and Vietnamese were consulted).
Periods in which searches were conducted	Initial corpus assembly and keyword searches: August 2026. Verification of the currency of the normative documents and gap-targeted searches: August 2026. Revision-stage searches (five additional references): October 2026. Exact day-level dates were not recorded.
Eligibility criteria and source hierarchy	As stated in Section 2, primary normative texts were preferred for definitions, sample bases, classification logic and limits; primary analytical or experimental studies were preferred for chemical compositions, volatile profiles, insect-associated changes, storage transformations, microbial associations, and sensory results; reviews and technical institutional sources were used to frame mechanisms or identify gaps where primary evidence was not available. Non-verifiable trade claims, unsupported sensory descriptors, and sources that did not identify their material or method sufficiently for the claim being made were excluded.
Selection process	All candidate sources were screened by the authors against the eligibility criteria above. Database retrieval was complemented by backward and forward citation chasing from the included publications. Normative texts were obtained directly from the issuing organizations. No independent dual screening was performed, and numbers of records retrieved, screened, and excluded were not recorded.
Composition of the final reference list	The revised reference list comprises primary normative and institutional documents (ISO and Codex standards; SCAA/SCA documents including the Coffee Value Assessment standards; ICE contract rules and rule submissions; national standards or regulatory instruments of thirteen producing countries and regions; ICO documents; EU legislation; and EFSA scientific opinion); peer-reviewed journal articles, which constitute the majority of entries; conference proceedings (ASIC colloquia); books and book chapters; and one industry technical document (the EM0011 defect sample-set booklet).
Elements of a fully systematic protocol that were not applied or recorded	Saved per-database search strings; PRISMA-type counts of records identified, screened, excluded, and included; independent dual screening; formal assessment of study quality or risk of bias. The resulting risk of literature omission and selection bias is acknowledged in Section 7.5.

## References

[1] (2004). Green Coffee–Defect Reference Chart, 2nd ed..

[2] (2025). Green Coffee–Olfactory and Visual Examination and Determination of Foreign Matter and Defects, 3rd ed..

[3] Specialty Coffee Association of America (2013). SCAA Arabica Green Coffee Defect Handbook, original ed. 2 April 2004, revised 26 April 2013.

[4] Toci A.T., Farah A. (2008). Volatile compounds as potential defective coffee beans’ markers. Food Chem..

[5] Toci A.T., Farah A. (2014). Volatile fingerprint of Brazilian defective coffee seeds: Corroboration of potential marker compounds and identification of new low quality indicators. Food Chem..

[6] Mancha Agresti P.D.C., Franca A.S., Oliveira L.S., Augusti R. (2008). Discrimination between defective and non-defective Brazilian coffee beans by their volatile profile. Food Chem..

[7] Sunarharum W.B., Williams D.J., Smyth H.E. (2014). Complexity of coffee flavor: A compositional and sensory perspective. Food Res. Int..

[8] Poisson L., Auzanneau N., Mestdagh F., Blank I., Davidek T. (2018). New insight into the role of sucrose in the generation of α-diketones upon coffee roasting. J. Agric. Food Chem..

[9] Poisson L., Schaerer A., Spreng S., Mestdagh F., Blank I., Davidek T. (2019). Generation of α-diketones and 4-hydroxy-2,5-dimethyl-3(2H)-furanone upon coffee roasting—Impact of roast degree on reaction pathways. J. Agric. Food Chem..

[10] Poisson L., Blank I., Dunkel A., Hofmann T., Folmer B. (2017). The chemistry of roasting—decoding flavor formation. The Craft and Science of Coffee.

[11] Farah A., Hii C.L., Borém F.M. (2019). Flavor development during roasting. Drying and Roasting of Cocoa and Coffee.

[12] Coffee Consulate (2026). Coffee Defects Sample Set EM0011: Companion Booklet to the Sample Set.

[13] ICE Futures U.S. Coffee “C” Rules, Chapter 8. https://www.ice.com/publicdocs/rulebooks/futures_us/8_Coffee.pdf.

[14] ICE Futures Europe Section GGGG–Contract Rules: ICE Futures Robusta Coffee Futures Contract. https://www.ice.com/publicdocs/contractregs/105_SECTION_GGGG.pdf.

[15] Ministério da Agricultura, Pecuária e Abastecimento (MAPA) (2003). Instrução Normativa nº 8, de 11 de Junho de 2003: Regulamento Técnico de Identidade e de Qualidade para a Classificação do Café Beneficiado Grão Cru.

[16] Comité Nacional de Cafeteros de Colombia (2016). Resolución 02 de 2016: Normas de Calidad para Café Verde en Almendra para Exportación.

[17] Instituto Nacional de Calidad (INACAL) (2021). GIP 101:2021–Guía de Implementación de NTP 209.027:2018.

[18] (2016). Café Verde–Especificaciones, Preparaciones y Evaluación Sensorial.

[19] (2011). Café Verde–Tabla de Referencia de Defectos.

[20] (2018). Café. Café Verde y Café Oro. Requisitos de Calidad.

[21] (2011). Norma Técnica Obligatoria Nicaragüense de Café Verde. Segunda Revisión.

[22] (1999). Green Coffee Beans–Specification.

[23] Government of Uganda (1994). The Coffee Regulations, 1994.

[24] Government of Tanzania (2013). The Coffee Industry Regulations, 2013.

[25] Biji Kopi. Badan Standardisasi Nasional: Jakarta, Indonesia, 2008.

[26] Cà Phê Nhân (Green Coffee). Viện Tiêu chuẩn Chất lượng Việt Nam: Hanoi, Vietnam, 2014.

[27] Coffee Industry Corporation Ltd (2016). Appendix 4–Coffee Parchment Standards, Factory Standards and Export Grades.

[28] (2005). Coffee and Coffee Products–Vocabulary, 4th ed..

[29] (2025). Green Coffee–Determination of Loss in Mass at 105 °C, 3rd ed..

[30] Specialty Coffee Association (2024). A System to Assess Coffee Value.

[31] International Coffee Organization (2004). Resolution No. 420: Coffee Quality-Improvement Programme.

[32] International Coffee Organization (2006). Harmonisation of ICC Resolution 420 with the ISO Green Coffee Defects Chart.

[33] (2024). Coffee Value Assessment: Sample Preparation and Tasting Mechanics.

[34] (2024). Coffee Value Assessment: Descriptive Assessment.

[35] (2024). Coffee Value Assessment: Affective Assessment.

[36] ICE Futures U.S. (2025). Submission No. 25-157: Listing of New Core Reference Futures Contract–Coffee “C” Metric Futures and Related Options Contracts.

[37] Bytof G., Hii C.L., Borém F.M. (2019). Flavor development during postharvest treatment of coffee: A holistic approach. Drying and Roasting of Cocoa and Coffee.

[38] Cao X., Wu H., Viejo C.G., Dunshea F.R., Suleria H.A.R. (2023). Effects of postharvest processing on aroma formation in roasted coffee—A review. Int. J. Food Sci. Technol..

[39] Geromel C., Ferreira L.P., Guerreiro S.M.C., Cavalari A.A., Pot D., Pereira L.F.P., Leroy T., Vieira L.G.E., Mazzafera P., Marraccini P. (2006). Biochemical and genomic analysis of sucrose metabolism during coffee (*Coffea arabica*) fruit development. J. Exp. Bot..

[40] Osorio Pérez V., Matallana Pérez L.G., Fernandez-Alduenda M.R., Álvarez Barreto C.I., Gallego Agudelo C.P., Montoya Restrepo E.C. (2023). Chemical composition and sensory quality of coffee fruits at different stages of maturity. Agronomy.

[41] Alves A. (1955). Melhoramento do cafeeiro. VIII–Novas determinações sôbre poliembrionia, sementes sem embrião e lojas dos frutos sem sementes em Coffea. Bragantia.

[42] Franca A.S., Oliveira L.S., Mendonça J.C.F., Silva X.A. (2005). Physical and chemical attributes of defective crude and roasted coffee beans. Food Chem..

[43] Mazzafera P. (1999). Chemical composition of defective coffee beans. Food Chem..

[44] Bytof G., Knopp S.-E., Kramer D., Breitenstein B., Bergervoet J.H.W., Groot S.P.C., Selmar D. (2007). Transient occurrence of seed germination processes during coffee post-harvest treatment. Ann. Bot..

[45] Selmar D., Bytof G., Knopp S.-E., Breitenstein B. (2006). Germination of coffee seeds and its significance for coffee quality. Plant Biol..

[46] Zhang S.J., De Bruyn F., Pothakos V., Torres J., Falconi C., Moccand C., Weckx S., De Vuyst L. (2019). Following coffee production from cherries to cup: Microbiological and metabolomic analysis of wet processing of *Coffea arabica*. Appl. Environ. Microbiol..

[47] Cardoso W.S., Agnoletti B.Z., de Freitas R., de Abreu Pinheiro F., Pereira L.L. (2021). Biochemical aspects of coffee fermentation. Quality Determinants in Coffee Production.

[48] Ruta L.L., Farcasanu I.C. (2021). Coffee and yeasts: From flavor to biotechnology. Fermentation.

[49] Peñuela-Martínez A.E., Osorio-Giraldo C.V., Buitrago-Zuluaga C., Medina-Rivera R.D. (2025). Development of fermentation strategies for quality mild coffee production (*Coffea arabica* L.) based on oxygen availability and processing time. Foods.

[50] Kramer D., Breitenstein B., Kleinwächter M., Selmar D. (2010). Stress metabolism in green coffee beans (*Coffea arabica* L.): Expression of dehydrins and accumulation of GABA during drying. Plant Cell Physiol..

[51] Kulapichitr F., Borompichaichartkul C., Suppavorasatit I., Cadwallader K.R. (2019). Impact of drying process on chemical composition and key aroma components of Arabica coffee. Food Chem..

[52] Peñuela-Martínez A.E., Sanz-Uribe J.R., Medina-Rivera R.D. (2023). Influence of drying air temperature on coffee quality during storage. Rev. Fac. Nac. Agron. Medellín.

[53] Selmar D., Bytof G., Knopp S.-E. (2008). The storage of green coffee (*Coffea arabica*): Decrease of viability and changes of potential aroma precursors. Ann. Bot..

[54] Moon S.A., Wongsakul S., Kitazawa H., Saengrayap R. (2022). Lipid oxidation changes of arabica green coffee beans during accelerated storage with different packaging types. Foods.

[55] Pardo E., Marín S., Ramos A.J., Sanchis V. (2005). Effect of water activity and temperature on mycelial growth and ochratoxin A production by isolates of Aspergillus ochraceus on irradiated green coffee beans. J. Food Prot..

[56] Akbar A., Medina A., Magan N. (2020). Resilience of Aspergillus westerdijkiae strains to interacting climate-related abiotic factors: Effects on growth and ochratoxin A production on coffee-based medium and in stored coffee. Microorganisms.

[57] Leitão A.L. (2019). Occurrence of ochratoxin A in coffee: Threads and solutions—A mini-review. Beverages.

[58] Johnson M.A., Ruiz-Diaz C.P., Manoukis N.C., Verle Rodrigues J.C. (2020). Coffee berry borer (*Hypothenemus hampei*), a global pest of coffee: Perspectives from historical and recent invasions, and future priorities. Insects.

[59] Silva S.A., Pereira R.G.F.A., Chalfoun S.M., Teixeira A.R. (2024). Physical and chemical attributes of beans damaged by the coffee berry borer at different levels of infestation. Bragantia.

[60] Carrión G., Bonet A. (2004). Mycobiota associated with the coffee berry borer (Coleoptera: Scolytidae) and its galleries in fruit. Ann. Entomol. Soc. Am..

[61] Silva S.A., Pereira R.G.F.A., Lira N.A., Glória E.M., Chalfoun S.M., Batista L.R. (2020). Fungi associated to beans infested with coffee berry borer and the risk of ochratoxin *A*. Food Control.

[62] Rini A.I.P., Wiranatha A.A.P.A.S., Yoga I.W.G.S. (2017). Pengaruh kadar biji pecah dalam penyangraian terhadap citarasa kopi Robusta Desa Pucak Sari, Buleleng, Bali. J. Rekayasa Dan Manaj. Agroindustri.

[63] Hu G.-L., Quan C.-X., Dai H.-P., Qiu M.-H. (2024). Characterization of defective coffee beans and blends differentiation based on 1H qNMR technique. Curr. Res. Food Sci..

[64] Farah A., de Paula Lima J. (2019). Consumption of chlorogenic acids through coffee and health implications. Beverages.

[65] Speer K., Kölling-Speer I. (2006). The lipid fraction of the coffee bean. Braz. J. Plant Physiol..

[66] Czerny M., Mayer F., Grosch W. (1999). Sensory study on the character impact odorants of roasted Arabica coffee. J. Agric. Food Chem..

[67] Czerny M., Grosch W. (2000). Potent odorants of raw Arabica coffee. Their changes during roasting. J. Agric. Food Chem..

[68] Flament I. (2002). Coffee Flavor Chemistry.

[69] Toledo P.R.A.B., Pezza L., Pezza H.R., Toci A.T. (2016). Relationship between the different aspects related to coffee quality and their volatile compounds. Compr. Rev. Food Sci. Food Saf..

[70] Ayseli M.T. (2025). The role of volatile and non-volatile compounds as quality indicators and marker candidates in coffee: A systematic review. J. Food Compos. Anal..

[71] Oliveira L.S., Franca A.S., Mendonça J.C.F., Barros-Júnior M.C. (2006). Proximate composition and fatty acids profile of green and roasted defective coffee beans. LWT Food Sci. Technol..

[72] Vasconcelos A.L.S., Franca A.S., Glória M.B.A., Mendonça J.C.F. (2007). A comparative study of chemical attributes and levels of amines in defective green and roasted coffee beans. Food Chem..

[73] Farah A., Monteiro M.C., Calado V., Franca A.S., Trugo L.C. (2006). Correlation between cup quality and chemical attributes of Brazilian coffee. Food Chem..

[74] Mendonça J.C.F., Franca A.S., Oliveira L.S., Nunes M. (2008). Chemical characterisation of non-defective and defective green arabica and robusta coffees by electrospray ionization-mass spectrometry (ESI-MS). Food Chem..

[75] Spadone J.-C., Takeoka G., Liardon R. (1990). Analytical investigation of Rio off-flavor in green coffee. J. Agric. Food Chem..

[76] Romano A., Navarini L., Lonzarich V., Bogialli S., Pastore P., Cappellin L. (2022). 2,4,6-Trichloroanisole off-flavor screening in green *Coffea arabica* by a novel Vocus NO+ CI-MS method: A study on green coffee from different geographical origins. J. Agric. Food Chem..

[77] Jackels S.C., Marshall E.E., Omaiye A.G., Gianan R.L., Lee F.T., Jackels C.F. (2014). GCMS investigation of volatile compounds in green coffee affected by potato taste defect and the Antestia bug. J. Agric. Food Chem..

[78] Cain C.N., Haughn N.J., Purcell H.J., Marney L.C., Synovec R.E., Thoumsin C.T., Jackels S.C., Skogerboe K.J. (2021). Analytical determination of the severity of potato taste defect in roasted East African Arabica coffee. J. Agric. Food Chem..

[79] Bade-Wegner H., Bendig I., Holscher W., Wollmann R. Volatile compounds associated with the over-fermented flavour defect. Proceedings of the 17th International Scientific Colloquium on Coffee (ASIC).

[80] Yeager S.E., Batali M.E., Guinard J.-X., Ristenpart W.D. (2023). Acids in coffee: A review of sensory measurements and meta-analysis of chemical composition. Crit. Rev. Food Sci. Nutr..

[81] Carvalho C.d.M., Moreira E.d.S., Zuim L., Paula M.C.d., Lima Filho T., Della Lucia S.M. (2023). Does the presence of defective beans in Arabica healthy coffee beans affect the sensory perception and acceptance of coffee beverages?. J. Sens. Stud..

[82] Dias E.C., Pereira R.G.F.A., Borém F.M., Mendes E., de Lima R.R., Fernandes J.O., Casal S. (2012). Biogenic amine profile in unripe Arabica coffee beans processed according to dry and wet methods. J. Agric. Food Chem..

[83] Specialty Coffee Association (n.d.). The Washed Arabica Green Coffee Defect Guide, Edition No. 3.

[84] Eira M.T.S., Amaral da Silva E.A., de Castro R.D., Dussert S., Walters C., Bewley J.D., Hilhorst H.W.M. (2006). Coffee seed physiology. Braz. J. Plant Physiol..

[85] van Mechelen D., Meulendijks D., Koumans M. (2025). Coffee bean characterization using terahertz sensing. Sensors.

[86] Silva C.F., Batista L.R., Schwan R.F. (2008). Incidence and distribution of filamentous fungi during fermentation, drying and storage of coffee (*Coffea arabica* L.) beans. Braz. J. Microbiol..

[87] Taniwaki M.H., Teixeira A.A., Teixeira A.R.R., Copetti M.V., Iamanaka B.T. (2014). Ochratoxigenic fungi and ochratoxin A in defective coffee beans. Food Res. Int..

[88] (2009). Code of Practice for the Prevention and Reduction of Ochratoxin A Contamination in Coffee.

[89] Bucheli P., Taniwaki M.H. (2002). Research on the origin, and on the impact of post-harvest handling and manufacturing on the presence of ochratoxin A in coffee. Food Addit. Contam..

[90] European Commission (2023). Commission Regulation (EU) 2023/915 of 25 April 2023 on maximum levels for certain contaminants in food and repealing Regulation (EC) No 1881/2006. Off. J. Eur. Union.

[91] Schrenk D., Bodin L., Chipman J.K., del Mazo J., Grasl-Kraupp B., Hogstrand C., Hoogenboom L.R., Leblanc J.-C., Nebbia C.S., EFSA Panel on Contaminants in the Food Chain (CONTAM) (2020). Risk assessment of ochratoxin A in food. EFSA J..

[92] Iamanaka B.T., Teixeira A.A., Teixeira A.R.R., Vicente E., Frisvad J.C., Taniwaki M.H., Bragagnolo N. (2014). Potential of volatile compounds produced by fungi to influence sensory quality of coffee beverage. Food Res. Int..

[93] Liato V., Aïder M. (2017). Geosmin as a source of the earthy-musty smell in fruits, vegetables and water: Origins, impact on foods and water, and review of the removing techniques. Chemosphere.

[94] Rendón M.Y., Garcia Salva T.J., Bragagnolo N. (2014). Impact of chemical changes on the sensory characteristics of coffee beans during storage. Food Chem..

[95] Scheidig C., Czerny M., Schieberle P. (2007). Changes in key odorants of raw coffee beans during storage under defined conditions. J. Agric. Food Chem..

[96] Błaszkiewicz J., Nowakowska-Bogdan E., Barabosz K., Kulesza R., Dresler E., Woszczyński P., Biłos Ł., Matuszek D.B., Szkutnik K. (2023). Effect of green and roasted coffee storage conditions on selected characteristic quality parameters. Sci. Rep..

[97] Walker H.E., Lehman K.A., Wall M.M., Siderhurst M.S. (2019). Analysis of volatile profiles of green Hawai’ian coffee beans damaged by the coffee berry borer (*Hypothenemus hampei*). J. Sci. Food Agric..

[98] Awgichew A., Nuguse R. (2024). Adaptation and performance evaluation of engine-driven coffee de-huller. Middle East J. Appl. Sci. Technol..

[99] Ghosh B.N. (1967). Studies on the effect of high temperature milling on the quality of Arabica coffee beans. J. Agric. Eng. Res..

[100] Machado M., Espírito Santo L., Machado S., Lobo J.C., Costa A.S.G., Oliveira M.B.P.P., Ferreira H., Alves R.C. (2023). Bioactive potential and chemical composition of coffee by-products: From pulp to silverskin. Foods.

[101] Bastian F., Pasilong T.P.B., Dirpan A., Hutabarat O.S., Rasyid I., Payung J.N.P.B. (2026). MMSE-GC-MS volatile organic compound profiling of twelve defect categories in Robusta coffee beans: Chemical markers and multivariate discrimination of quality defects. Food Chem..

[102] Turck D., Bohn T., Castenmiller J., De Henauw S., Hirsch-Ernst K.I., Maciuk A., Mangelsdorf I., McArdle H.J., Naska A., EFSA Panel on Nutrition, Novel Foods and Food Allergens (NDA) (2022). Safety of dried coffee husk (cascara) from *Coffea arabica* L. as a novel food pursuant to Regulation (EU) 2015/2283. EFSA J..

[103] Stevens J.C., Cain W.S., Burke R.J. (1988). Variability of olfactory thresholds. Chem. Senses.

[104] Cantergiani E., Brévard H., Krebs Y., Feria-Morales A., Amadò R., Yeretzian C. (2001). Characterisation of the aroma of green Mexican coffee and identification of mouldy/earthy defect. Eur. Food Res. Technol..

[105] Becker R., Döhla B., Nitz S., Vitzthum O.G. Identification of the ‘peasy’ off-flavour note in Central African coffees. Proceedings of the 12th International Scientific Colloquium on Coffee (ASIC).

[106] Gueule D., Fourny G., Ageron E., Le Flèche-Matéos A., Vandenbogaert M., Grimont P.A.D., Cilas C. (2015). Pantoea coffeiphila sp. nov., cause of the ‘potato taste’ of Arabica coffee from the African Great Lakes region. Int. J. Syst. Evol. Microbiol..

[107] Hale A.R., Ruegger P.M., Rolshausen P., Borneman J., Yang J. (2022). Fungi associated with the potato taste defect in coffee beans from Rwanda. Bot. Stud..

[108] Bigirimana J., Mubashankwaya I., Niyibigira T., Nzeyimana F. (2025). Mechanical damage causes potato taste defect in coffee without antestia bug infestations: Evidence from experimental data. Int. J. Fruit Sci..

[109] Cain C.N., Gaida M., Stefanuto P.-H., Focant J.-F., Synovec R.E., Jackels S.C., Skogerboe K.J. (2024). Investigating sensory-classified roasted arabica coffee with GC × GC-TOFMS and chemometrics to understand potato taste defect. Microchem. J..

[110] Agudelo Gómez D., Oliveros Tascón C.E., Estrada Estrada J.H., Álvarez Mejía F., Sanz Uribe J.R. (2008). Desarrollo de un separador de objetos duros en el café a partir de las vibraciones producidas en placas delgadas. Rev. Fac. Nac. Agron. Medellín.

[111] (2008). Green Coffee–Preparation of Samples for Use in Sensory Analysis, 2nd ed..

[112] Hong S., Zhang D., Chi H., He J., Guo X., Fu H., Wang L. (2026). Automated detection of defective coffee beans based on improved YOLOv10 framework. Curr. Res. Food Sci..

[113] Sandoval-Gonzalez O., Manrique-Santos D., Cruz-Jarquin D., Portillo-Rodriguez O., Gonzalez-Sanchez B., Landeta-Escamilla O., Aguila-Rodriguez G. (2026). Dual-sided green coffee bean defect inspection using a mechatronic system with AI-powered computer vision. Agriculture.

[114] Yang N., Liu C., Liu X., Degn T.K., Munchow M., Fisk I. (2016). Determination of volatile marker compounds of common coffee roast defects. Food Chem..

